# Supplementary material for: A guilt-free strategy increases self-reported non-compliance with COVID-19 preventive measures: Experimental evidence from 12 countries
Source: PLoS One. 2021 Apr 21;16(4):e0249914. doi: 10.1371/journal.pone.0249914 (PMC8059824; doi:10.1371/journal.pone.0249914)
Supplement: S1 File — (PDF) [file pone.0249914.s001.pdf]

S1 File

A Guilt-Free Strategy Increases Self-Reported Non-Compliance with COVID-19 Preventive Measures: Experimental Evidence from 12 Countries

PLOS One

Appendix A: Description of the samples and national contexts

Table A.1. Description of the samples

Country	Dates on the field	Number of observations
Australia	June, 15th – 19th	1,003
Austria	June, 23rd – 27th	1,011
Brazil	June, 23rd – 28th	1,000
France	June, 22nd – 25th	1,006
Germany	June, 23rd – 27th	1,004
Italy	June, 24th – 27th	1,003
New Zealand	June, 23rd – 28th	1,000
Poland	June, 24th – 27th	1,014
Spain	July, 6th – 10th	961
Sweden	June, 23rd – 27th	1,017
United Kingdom	June, 21st – 24th	1,014
United States	June, 16th – 22nd	1,001
Canada*	June, 16th – 24th	1,002

*Note. *: used for robustness checks only.*

Table A.2. Population and mortality rate across countries

Country	Population (million)	COVID-19 Mortality rate per 100 000 inhabitants (June 15)
Australia	25.36	0.40
Austria	8.87	7.63
Brazil	211.05	20.82
France	67.06	43.80
Germany	83.13	10.59
Italy	60.29	57.00
New Zealand	4.91	0.44
Poland	37.97	3.30
Spain	47.07	57.64
Sweden	10.28	47.55
US	328.24	36.19
United Kingdom	66.83	58.94
Canada*	37.58	21.88

*Note: Source for the population is the World Bank and the Mortality rates and computed using John Hopkins Coronavirus Resource Center data (accessible on github). *: used for robustness checks only.*

Table A.3. COVID-19 Preventive Measures across countries

	Mandatory use of masks in shops and public transportation		Mandatory physical distancing		Stay at home requirement	
	Policy	Scope	Policy	Scope	Policy	Scope
**Australia**	No	National	Yes	National	No	National
**Austria**	In public transportation	National	Yes	National	No	National
**Brazil**	In shops and/or public transportation	In specific areas in state or in whole state	Yes	In specific areas in state or in whole state	Require not leaving house with exceptions for daily exercise, grocery shopping, and 'essential' trips	In specific areas
**France**	In public transportation	National	Yes	National	No	National
**Germany**	In public transportation and shops	National (agreement of federal and state governments) / all states except Berlin	Yes	National	No	National
**Italy**	In shops and public transportation	National	Yes	National	Recommend not leaving house	National
**New Zealand**	No	National	No	National	No	National
**Poland**	In shops and public transportation	National	yes	National	No	National
**Spain**	In shops and restaurants; also in all public spaces when social distancing not possible	Specific regions / national	Yes	National	Recommend not leaving house	In specific regions
**Sweden**	No	National	No	National	Recommend not leaving house	National
**United Kingdom**	In public transportation	National	Yes	National	Recommend not leaving house	In specific areas
**United States**	In shops and/or public transportation	In specific areas in state or in whole state	Yes	In specific areas in state or in whole state	Require not leaving house with exceptions for daily exercise, grocery shopping, and 'essential' trips	In specific areas in state or in whole state
**Canada***	For air passengers / in shops and public transportation	National / in some provinces or in specific areas of some provinces	Yes	National	Recommend not leaving house	National

Source: https://github.com/amel-github/covid19-interventionmeasures; https://masks4all.co/what-countries-require-masks-in-public; https://airtable.com/shrxEdbaBJIQwMUgA/tbl5o6qUd54BL9wkw; https://www.bsg.ox.ac.uk/research/research-projects/coronavirus-government-response-tracker; https://kubinec.shinyapps.io/coronanet; and further authors' corroborations with official and media sources.

Appendix B: Descriptive statistics, by country

Figure B.1. Non-compliance with preventive measures

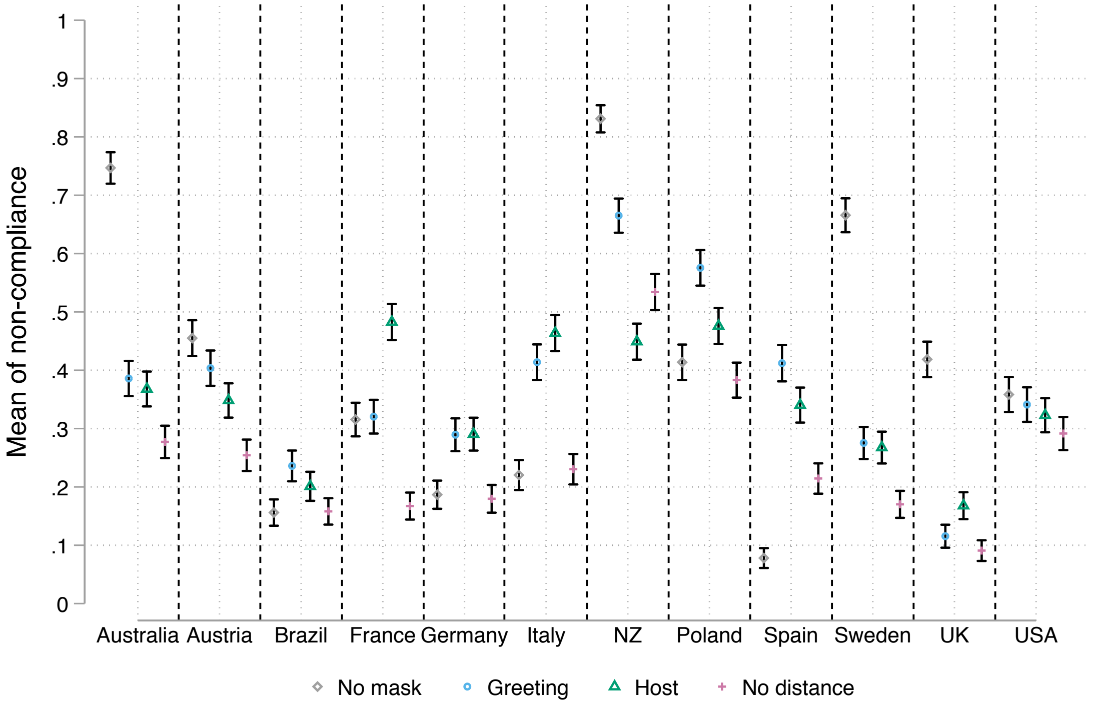

Note: Means are shown for each item in each country, with 95% confidence intervals included.

Figure B.2. Non-compliance, by guilt-free answer choices

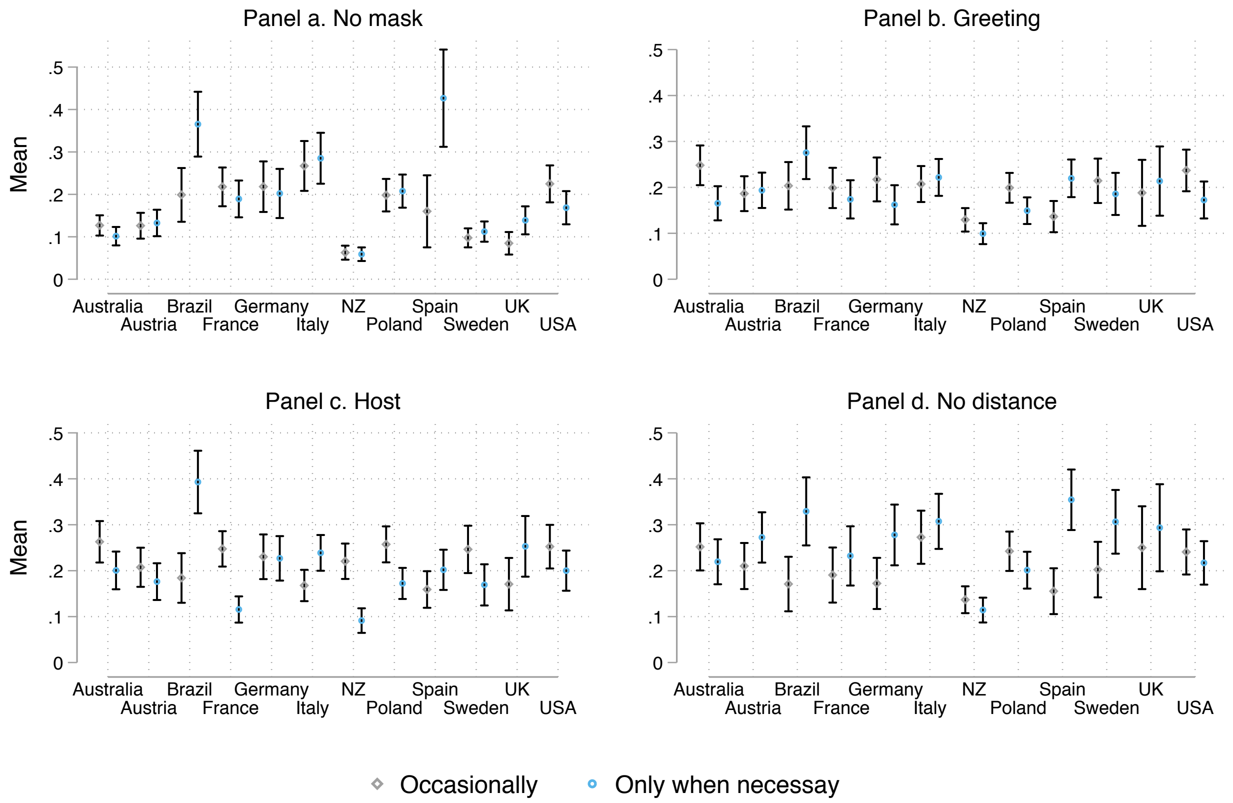

Note: Means are shown for each item and guilt-free answer choices, with 95% confidence intervals included.

Table B.1. Descriptive statistics for age, gender and education.

	Australia		Austria		Brazil		France		Germany		Italy	
	Mean	Std. Dev.	Mean	Std. Dev.	Mean	Std. Dev.	Mean	Std. Dev.	Mean	Std. Dev.	Mean	Std. Dev.
Age	46.64	14.43	47.30	16.47	41.33	15.01	3.97	1.70	51.02	15.91	47.68	16.48
Gender	0.52	0.50	0.47	0.50	0.52	0.50	0.49	0.50	0.51	0.50	0.53	0.50
Education	0.46	0.27	0.49	0.22	0.63	0.15	0.72	0.31	0.62	0.28	0.57	0.19
	NZ		Poland		*Spain*		Sweden		UK		USA	
Age	48.37	15.58	43.96	15.37	48.27	17.54	51.45	16.14	46.65	16.03	46.41	17.90
Gender	0.53	0.50	0.53	0.50	0.52	0.50	0.49	0.50	0.51	0.50	0.53	0.50
Education	0.55	0.26	0.71	0.26	0.54	0.19	0.65	0.34	0.61	0.29	0.58	0.21

Note: For gender, female=1

Appendix C. Regression tables

Table C.1. Regressions outputs for panel a of Figure 2

	Face mask	Greeting	Host	Distance
Gender (1=Female)	-0.14	-0.08	-0.03	-0.22
	(0.05)	(0.06)	(0.06)	(0.07)
Treatment	0.40	0.48	0.76	0.78
	(0.05)	(0.05)	(0.06)	(0.06)
Gender × Treatment	-0.05	0.09	-0.07	0.02
	(0.07)	(0.08)	(0.08)	(0.09)
Constant	-0.50	-0.77	-0.99	-1.44
	(0.04)	(0.04)	(0.04)	(0.05)
Observations	12026	12026	12023	12028

Note: Logistic regression coefficients with standard errors in parentheses.

Table C.2. Regressions outputs for panel b of Figure 2

	Face mask	Greeting	Host	Distance
Age	-0.0089	-0.0284	-0.0168	-0.0316
	(0.0017)	(0.0018)	(0.0018)	(0.0022)
Treatment	0.5295	0.6885	0.9339	0.8246
	(0.1151)	(0.1200)	(0.1202)	(0.1327)
Age × Treatment	-0.0032	-0.0028	-0.0042	0.0002
	(0.0023)	(0.0025)	(0.0025)	(0.0029)
Constant	-0.1536	0.5044	-0.2233	-0.1353
	(0.0822)	(0.0859)	(0.0881)	(0.1009)
Observations	12026	12026	12023	12028

Note: Logistic regression coefficients with standard errors in parentheses.

Table C.3. Regressions outputs for panel c of Figure 2

	Face mask	Greeting	Host	Distance
Education	-0.24	0.22	0.24	0.21
	(0.10)	(0.11)	(0.11)	(0.13)
Treatment	0.23	0.63	0.69	0.71
	(0.09)	(0.09)	(0.10)	(0.11)
Education × Treatment	0.24	-0.17	0.05	0.12
	(0.14)	(0.14)	(0.15)	(0.17)
Constant	-0.44	-0.94	-1.15	-1.68
	(0.07)	(0.07)	(0.07)	(0.09)
Observations	11946	11946	11943	11948

Note: Logistic regression coefficients with standard errors in parentheses.

Appendix D. Robustness checks

Figure D.1. Replication of Figure 1 with weights

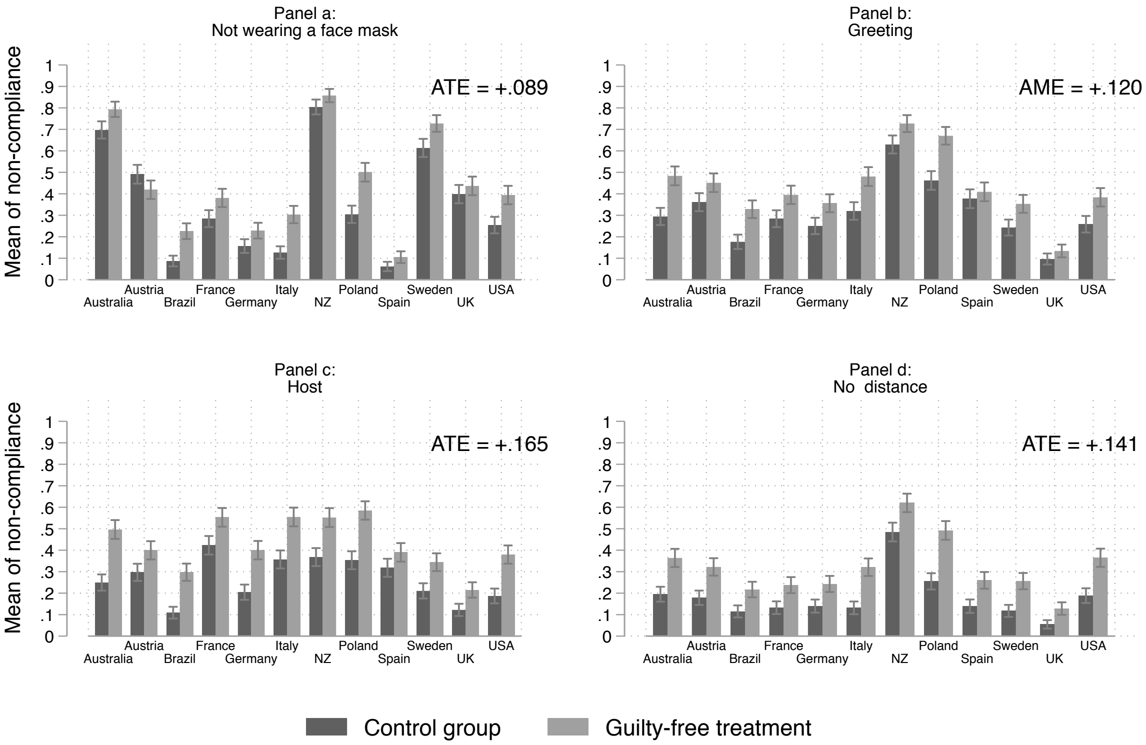

Note: Means of non-compliance are shown with 95% confidence intervals included. ATE=Average treatment effect.

Figure D.2. Replication of Figure 2 with weights

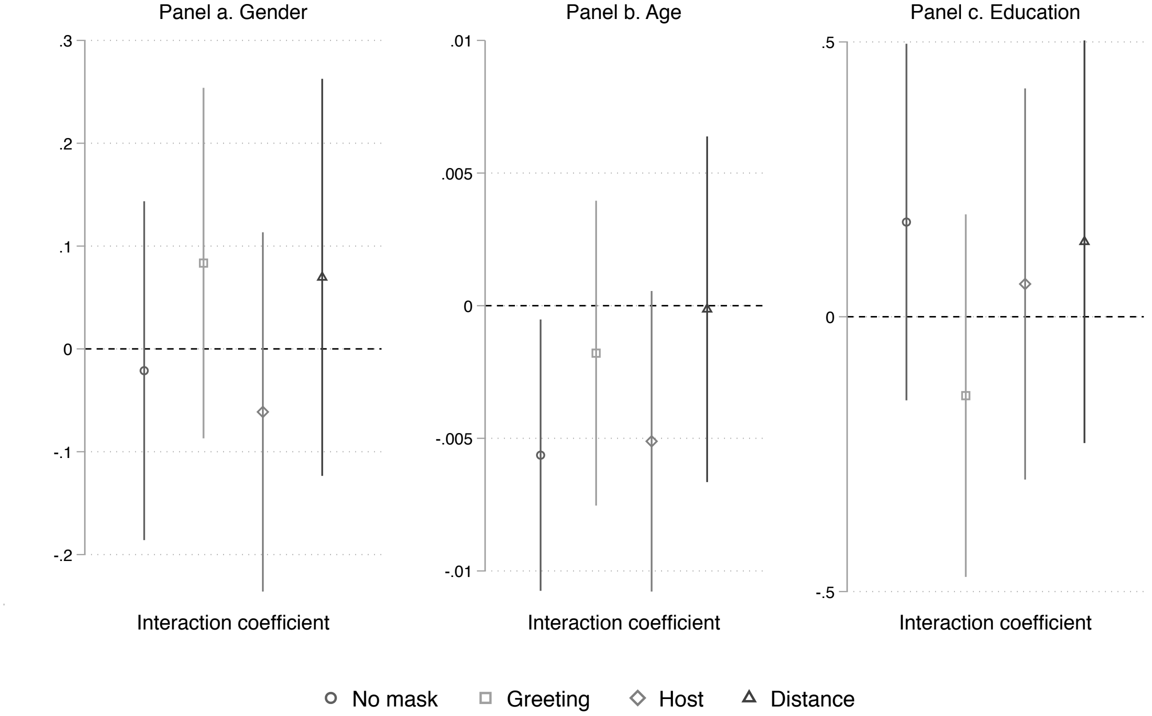

Note: The values of the interaction coefficients with 95% confidence intervals included.

Figure D.3. Average treatment effects controlling for country fixed effects

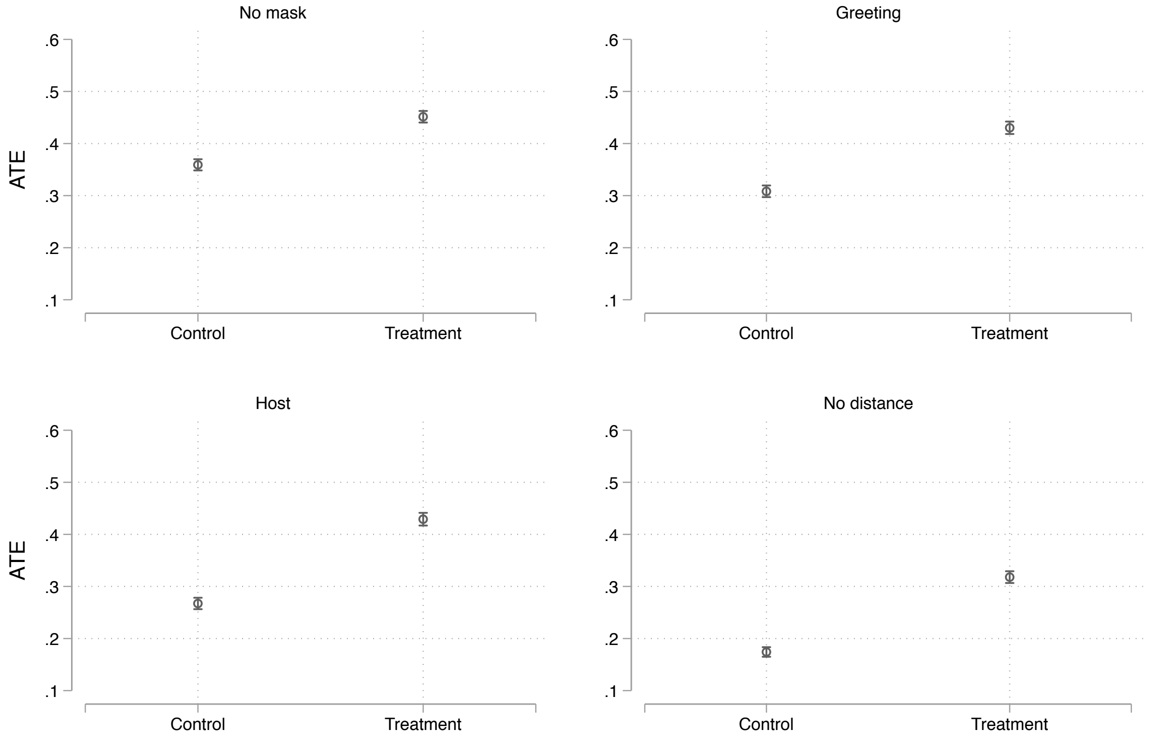

Note: Average treatment effects are estimated from Table D.1. and are shown with 95% confidence intervals included.

Table D.1. Regressions models with country fixed effects

Dependent variable =	No mask	Greeting	Host	No distance
Guilt-free treatment	0.49	0.58	0.76	0.86
	(0.04)	(0.04)	(0.04)	(0.05)
Austria	-1.28	0.07	-0.09	-0.12
	(0.10)	(0.09)	(0.09)	(0.10)
Brazil	-2.81	-0.72	-0.86	-0.74
	(0.11)	(0.10)	(0.10)	(0.11)
France	-1.88	-0.29	0.49	-0.67
	(0.10)	(0.09)	(0.09)	(0.11)
Germany	-2.58	-0.44	-0.37	-0.58
	(0.11)	(0.10)	(0.10)	(0.11)
Italy	-2.38	0.12	0.41	-0.26
	(0.11)	(0.09)	(0.09)	(0.10)
New Zealand	0.52	1.18	0.35	1.15
	(0.11)	(0.09)	(0.09)	(0.10)
Poland	-1.45	0.78	0.46	0.50
	(0.10)	(0.09)	(0.09)	(0.10)
Spain	-3.60	0.11	-0.13	-0.36
	(0.14)	(0.09)	(0.10)	(0.11)
Sweden	-0.40	-0.51	-0.48	-0.64
	(0.10)	(0.10)	(0.10)	(0.11)
United Kingdom	-1.43	-1.59	-1.09	-1.38
	(0.10)	(0.12)	(0.11)	(0.13)
United States	-1.69	-0.19	-0.19	0.09
	(0.10)	(0.09)	(0.10)	(0.10)
Constant	0.85	-0.76	-0.94	-1.43
	(0.08)	(0.07)	(0.07)	(0.08)
Observations	12026	12026	12023	12028

Note. Logistic regression predicting non-compliance. Standard errors in parentheses. The reference category for the countries is Australia.

Figure D.4. Prohibited and non-prohibited (placebo) items

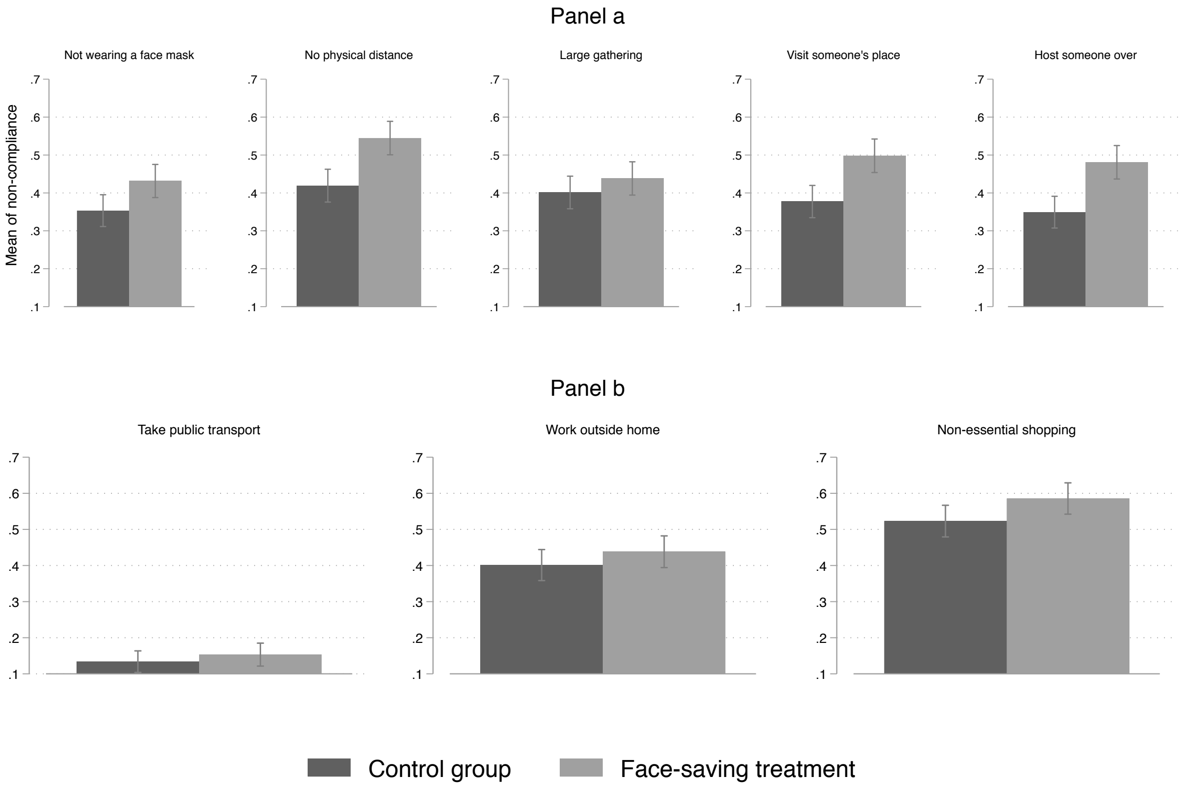

Note: Means of non-compliance are shown with 95% confidence intervals included.

Figure D.5. Comparison with other estimation techniques

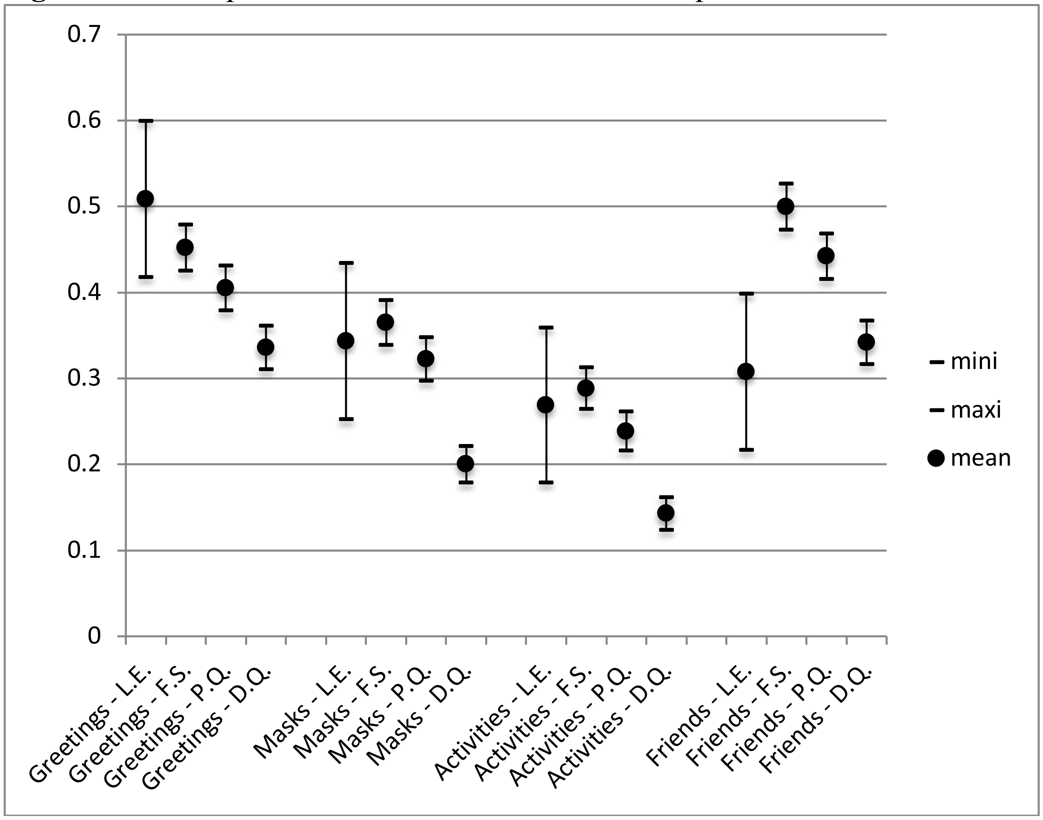

Note: Means with 95% confidence intervals are shown. D.Q. indicates estimation from the "dichotomous" question format; P.Q. indicates estimation from the "polytomous" question format; F.S. indicates estimation from the "face saving" question format; L.E. indicates estimation from the list-experiment estimation strategy. Masks refers to the following items: Go shopping or take public transportation without a face mask or taking it off during it; Greetings refers to the following items: Meet friends, family or colleagues greeting them by shaking hands, hugging or kissing; Activities refers to the following items: Participate in social activities (work, sport, religious ceremony…) without respecting physical distancing. Friends refers to the following items: Have a group of friends or family over at your place without respecting physical distancing.

Data: We ran a quota-based online survey in France that was in field from July 9-19th. As nearly all surveys in France, sampling was done using a quota method based on age, gender, occupation, regions and type of residential area. The sample is designed to be representative of the electorate's features in each of the 12 metropolitan French regions. 10,686 respondents answered our questionnaire.

The same four items included in the survey were:

- Go shopping or take public transportation without a face mask or taking it off during it;
- Meet friends, family or colleagues greeting them by shaking hands, hugging or kissing;
- Have a group of friends or family over at your place without respecting physical distancing;
- Participate in social activities (work, sport, religious ceremony…) without respecting physical distancing.

We used four different strategies to address the issue of social desirability. Three approaches focusing on the question wording and answer options, and a list-experimental approach:

- First, a set of 1343 respondents were exposed to the following "dichotomous" question wording discussed by Daoust et al. (2020): "Have you done any of the following activities in the last week?". The response categories were (1) yes, (2) no.
- Second we administered to another group of 1337 respondents the "polytomous" question used by Munzert & Selb (2020) : "How often have you done the following activities in the last week?". The response categories were (1) daily, (2) several times, (3) once, (4) never.
- The third type of questions included is the "face-saving" question proposed by Daoust et al. (2020): "Some people have altered their behaviour since the beginning of the pandemic, while others have continued to pursue various activities. Some may also want to change their behaviour but cannot do so for different reasons. Have you done any of the following activities in the last week?". 1345 respondents were to choose between the following categories: (1) Yes, (2) Occasionally, (3) Only when necessary, (4) No.
- Finally, we also embedded a list experiment (Becher 2020; Larsen 2020; Munzert & Selb 2020) in this survey. Faced with a list of items, a fourth group of respondents were asked how many things they had done last week, but not which specific ones. Respondents were randomly assigned to either the control group (N=1334) or one of the four treatment groups (N=1332; N=1331; N=1335; N=1329). The treated groups received an additional item capturing the violation of the social distancing norm. The control group received a list of four behaviours that are generally permissible under existing health recommendations. The question reads as follow: "How many of these things have you done last week? You do not need to tell me which ones you have done, just how many." The following four activities were listed: I went to the doctor or to the hospital; I watched a movie or TV show online using a streaming service (e.g., Amazon, Netflix); I exercised outdoors; I ordered food using an online delivery service. In addition to these four items, the treated groups received one of the four more sensitive items we study. The estimated level of non-compliance was obtained from the simple differences-in-means.

Turning to the results from these different approaches, as shown in Figure D.5., for 3 out of 4 items the level of non-compliance estimated by the face-saving question (the estimates denoted by F.S.) is in the 95% confidence interval of the level of non-compliance estimated by the list experiment (the estimates denoted by L.E.). For one item, the face-saving estimate is 4.5 points below and for both other ones it is 2 points higher. For the items related to mask-wearing, greetings and social-distancing in activities, estimates from the dichotomous questions are systematically below the list-experiment estimates and beyond its 95% confidence intervals. The "face-saving" treatment does not seem to induce a systematic experimenter demand effect that would inflate non-compliance, at least compared with an alternative measurement strategy - list experiment – that is well known for decreasing desirability bias (Blair and Imai 2012) and does not appear prone to any experimenter demand effect due to the lack of any associated framing.

The pattern described above differs only for non-compliance in social distancing while hosting friends. In this instance, the list experiment estimate is the lowest. Only the estimate from the dichotomous question does not statistically differ from it. Both the estimates of the face-saving and polytomous questions are significantly higher. The polytomous question is devoid of any framing in the wording of the question. Therefore, even if we have no explanation for this

idiosyncratic pattern, it is unlikely that the higher estimate produced by the face-saving question relative to the list experiment is due to a potential experimenter demand effect induced by the face-saving treatment.

Overall, even if this experiment only includes France, it brings no evidence of an experimenter demand effect that would systematically bias our estimates.

Table D.2. Romano-Wolf test for the "No Mask" item

	Model p-value	Resample p-value	Romano-Wolf p-value
Australia	0.0039	0.0050	0.0200
Austria	0.0156	0 0230	0.0509
Brazil	0.0000	0.0010	0.0010
France	0.0002	0.0020	0.0030
Germany	0.0074	0.0060	0.0320
Italy	0.0000	0.0010	0.0010
New Zealand	0.0600	0.0619	0.1249
Poland	0.0000	0.0010	0.0010
Spain	0.0001	0.0010	0.0030
Sweden	0.0000	0.0010	0.0020
United Kingdom	0.1295	0.1518	0.1518
United States	0.0000	0.0010	0.0010

Note: Default options from the rwolf *package was used, with the number of repetitions set at 1000.*

Table D.3. Romano-Wolf test for the "Greeting" item

	Model p-value	Resample p-value	Romano-Wolf p-value
Australia	0.0000	0.0010	0.0010
Austria	0.0071	0.0080	0.0210
Brazil	0.0000	0.0010	0.0010
France	0.0004	0.0010	0.0010
Germany	0.0001	0.0010	0.0010
Italy	0.0000	0.0010	0.0010
New Zealand	0.0002	0.0010	0.0010
Poland	0.0000	0.0010	0.0010
Spain	0.0132	0.0150	0.0260
Sweden	0.0004	0.0010	0.0010
United Kingdom	0.0380	0.0360	0.0360
United States	0.0001	0.0010	0.0010

Note: Default options from the rwolf *package was used, with the number of repetitions set at 1000.*

Table D.4. Romano-Wolf test for the "Host" item

	Model p-value	Resample p-value	Romano-Wolf p-value
Australia	0.0000	0.0010	0.0010
Austria	0.0035	0.0050	0.0050
Brazil	0.0000	0.0010	0.0010
France	0.0001	0.0010	0.0020
Germany	0.0000	0.0010	0.0010
Italy	0.0000	0.0010	0.0010
New Zealand	0.0000	0.0010	0.0010
Poland	0.0000	0.0010	0.0010
Spain	0.0002	0.0010	0.0030
Sweden	0.0000	0.0010	0.0010
United Kingdom	0.0000	0.0010	0.0010
United States	0.0000	0.0010	0.0010

Note: Default options from the rwolf *package was used, with the number of repetitions set at 1000.*

Table D.5. Romano-Wolf test for the "Social Distance" item

	Model p-value	Resample p-value	Romano-Wolf p-value
Australia	0.0000	0.0010	0.0010
Austria	0.0000	0.0010	0.0010
Brazil	0.0000	0.0010	0.0010
France	0.0000	0.0010	0.0010
Germany	0.0000	0.0010	0.0010
Italy	0.0000	0.0010	0.0010
New Zealand	0.0000	0.0010	0.0010
Poland	0.0000	0.0010	0.0010
Spain	0.0000	0.0010	0.0010
Sweden	0.0000	0.0010	0.0010
United Kingdom	0.0000	0.0010	0.0010
United States	0.0000	0.0010	0.0010

Note: Default options from the rwolf *package was used, with the number of repetitions set at 1000.*
