# Supplementary material for: A guilt-free strategy increases self-reported non-compliance with COVID-19 preventive measures: Experimental evidence from 12 countries
Source: PLoS One. 2021 Apr 21;16(4):e0249914. doi: 10.1371/journal.pone.0249914 (PMC8059824; doi:10.1371/journal.pone.0249914)
Supplement: Attachment — Submitted filename: Response to Reviewers.pdf [file pone.0249914.s002.pdf]

Face-Saving Strategies Increase Self-Reported Non-Compliance with COVID-19 Preventive Measures: Experimental Evidence from 12 Countries

PLOS ONE

PONE-D-20-32568

We are grateful for the opportunity to submit a revised version of our paper "Face-Saving Strategies Increase Self-Reported Non-Compliance with COVID-19 Preventive Measures: Experimental Evidence from 12 Countries" and we thank the editor and reviewers for their careful reading of our manuscript. In revising our paper, we have taken into account the reviewers' comments and we have implemented a series of revisions. We believe that doing so has improved our paper.

In this memo, we detail how we have implemented our changes in the revised version of our paper. The reviewers' comments are copied in this note (in blue font), and they are followed by our responses (in black font, and preceded by three asterisks (***)).

Reviewer #1

The paper is written in a clear way and addresses an important question. Here are some comments:

***We thank R1 for their careful reading and helpful review. Below, we detail how we revised our manuscript in order to incorporate their comments.

-The authors write (page 12), "among several important subgroups of the population including gender, age and education". This implies that subgroups other than gender, age and education have been checked, but there is no evidence of that in the paper. Please, clarify.

***We fully agree with R1 that the sentence was oddly worded and thank the reviewer for spotting this. We now clarify that we only explored heterogeneity based on gender, age and education as follows:

> "*Moreover, the impact of the treatment effect is fairly homogenous. As shown in Figure 2, it does not seem to be conditioned by peoples' gender, age or level of education.*"

-The authors write (page 11), "The results for potential heterogeneous effects across subgroups based on gender, age and education are quite clear: overall there is no substantial moderation effect. The interaction coefficients never reach statistical significance at p<.05." Then, at page 12, they write, referring to the weighted dataset "One interaction reaches p<.05 for age but the single significant interaction for education now fails to pass that threshold". The two sentences appear to be in contradiction with each other.

***Again, we thank R1 for spotting this lack of clarity. When discussing Figure D.2 (from the Appendix) on page 12, we mistakenly mentioned that one interaction coefficient in Figure 2 reached significance while there was none. We corrected this error. None of the coefficients reached statistical significance in Figure 2. In contrast, one does so in Figure D.2, which we now clarify as follows:

> "*One interaction out of twelve reaches* p*<.05, that is, the interaction between age and treatment for the 'no mask' behaviour.*"

-In the discussion, the authors should go back to the results by Munzert and Selb (2020) on Germany and discuss why the findings differ.

***This is a fair point. In the revised version of our manuscript, we discuss previous experimental studies on this topic in more detail, including the relevant piece mentioned by R1. Our reading of Munzert and Selb (2020) is that they argue that a list experimental technique may not reduce social desirability bias as much as often thought in the literature, at least when it comes to non-pharmaceutical interventions in the pandemic. This does not imply that there is no social desirability bias. In fact, their results show that the prevalence of the undesirable behavior is higher when using a multivarlue response option compared to a simple yes-no question or the list experiment. It should also be noted that Munzert and Selb (2020) explicitly discuss the possibility that face-saving strategies might be a "valuable alternative" (p. 207). Our paper tests this conjecture comparatively. Below, we detail how we integrated this discussion in the revised version of our manuscript:

> "*Focusing on the current pandemic context, some of the first experimental studies of measurement strategies to reduce social desirability bias found limited evidence of social desirability bias when employing the list experimental technique (Larsen et al. 2020; Munzert and Selb 2020). Munzert and Selb (2020) report a difference in the prevalence of not following social distancing between the treated and untreated group of 6 percentage points that is "barely significant at conventional levels due to the vast measurement error of the list estimate." (p. 206) They also find that the prevalence of the undesirable behavior is higher when using multivalue response option compared to either a simple yes-no question or the list experimental estimate. This shows that dichotomous direct question should not be taken at face value. Discussing these findings, Munzert and Selb (2020) suggest that face-saving strategies employed by Daoust et al. (2020) might be a "valuable alternative" (p. 207). In this research, we test this face-saving strategy using a comparative experimental design and our results strengthen this conclusion.*"

-My main concern about the methodology adopted by the authors is that they compare a treatment with 2 options (yes/no) to a face-saving treatment with 4 options (yes/occasionally/only when necessary/no), where 3 (yes/occasionally/only when necessary) correspond to the single "yes" option in the direct question. The issue is that, if a subset of the sample answers randomly, then this will inflate the positive answers in the face-saving treatment, as there is a 75% probability that a random answer is "yes/occasionally/only when necessary" vs

a 50% probability that a random answer is "yes" in the direct question. This would generate spurious evidence of social desirability bias. The authors should address this concern.

***This is a fair point, and our answer is threefold. First, to the extent that random answers might be driving the results, this should probably come mostly from inattentive or low-attentive respondents. While we cannot know for sure how many survey respondents were inattentive, it is likely that they are not very numerous. For example, Maniaci and Rogge (2014) measured inattention in different ways (looking at the longest string of identical answer, self-reported careless measures, time completion of the survey, etc.) and found proportions of inattentive respondents ranging from 3 to 9% across their several US samples. Moreover, Anduiza and Galais' (2017) work included four different Spanish samples among which the rates of inattentive respondents were between 4.6-8.5%.

Given that respondents who are at risk of providing random answers (and thus inflating the non-compliers due to the increase in the number of answer choices) are the "low-quality" respondents, we would ideally show that our findings hold when excluding those inattentive people. Unfortunately, the surveys did not include attention checks or direct questions that allow identifying them directly. However, education is known to be systematically related to low attention in survey participation. Building on this insight, we could conceive of levels of education as a proxy for respondents' average attention levels. If randomness, driven by inattentive respondents, is mainly responsible for the effects of our experimental treatments, we should see that effects are systematically stronger among lower-educated relative to higher educated respondents in the samples. We divided respondents with a level of education higher than the median category from each country (versus the rest of the respondents). Supplementary analyses, that are reported in Figures R1 to R4, do not offer evidence that is in line with such a pattern. To be more precise, the differences in the effects among the least and the most educated are generally fairly similar, and we find no indications of the effects being systematically stronger among the lesser educated either.

Figure R.1. Distributions of guilt-free answer choices among the least and most educated, across countries: 'No mask' item

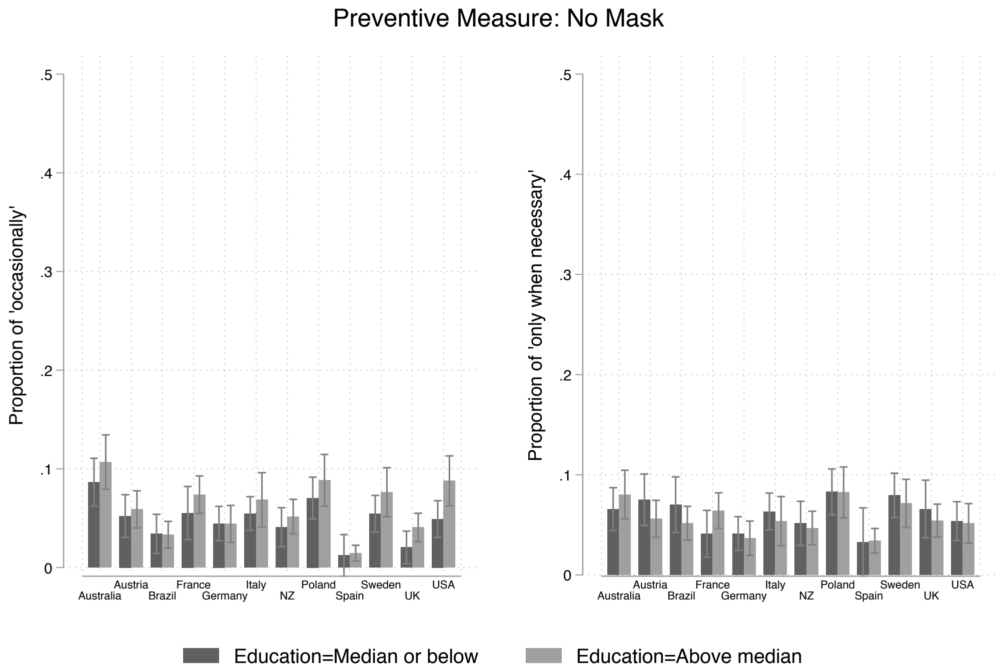

Figure R.2. Distributions of guilt-free answer choices among the least and most educated, across countries: 'Greeting' item

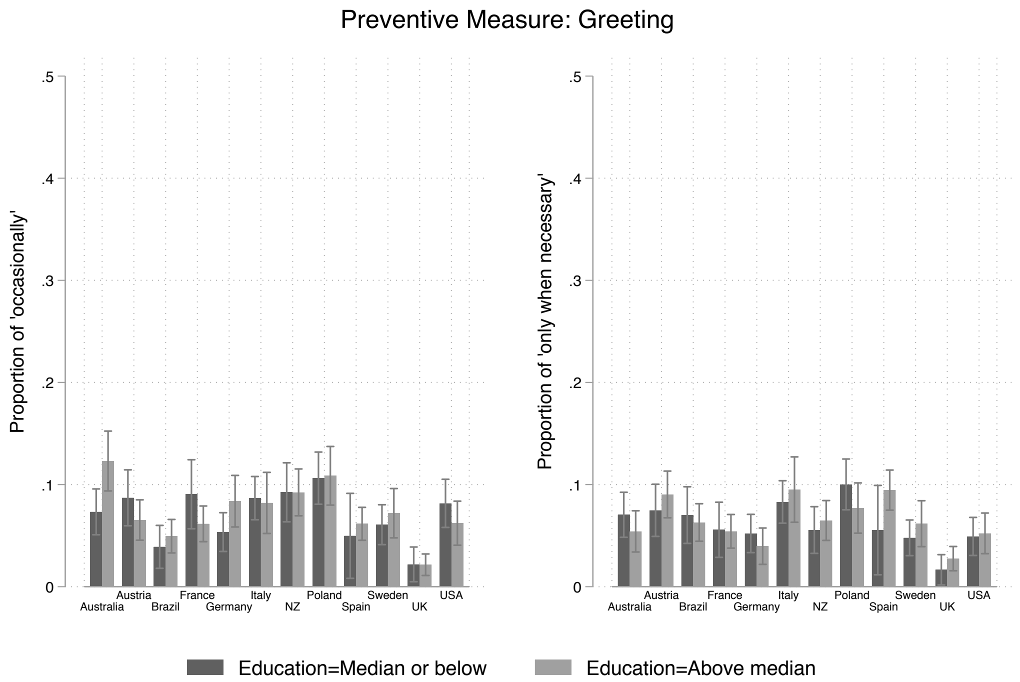

Figure R.3. Distributions of guilt-free answer choices among the least and most educated, across countries: 'Host' item

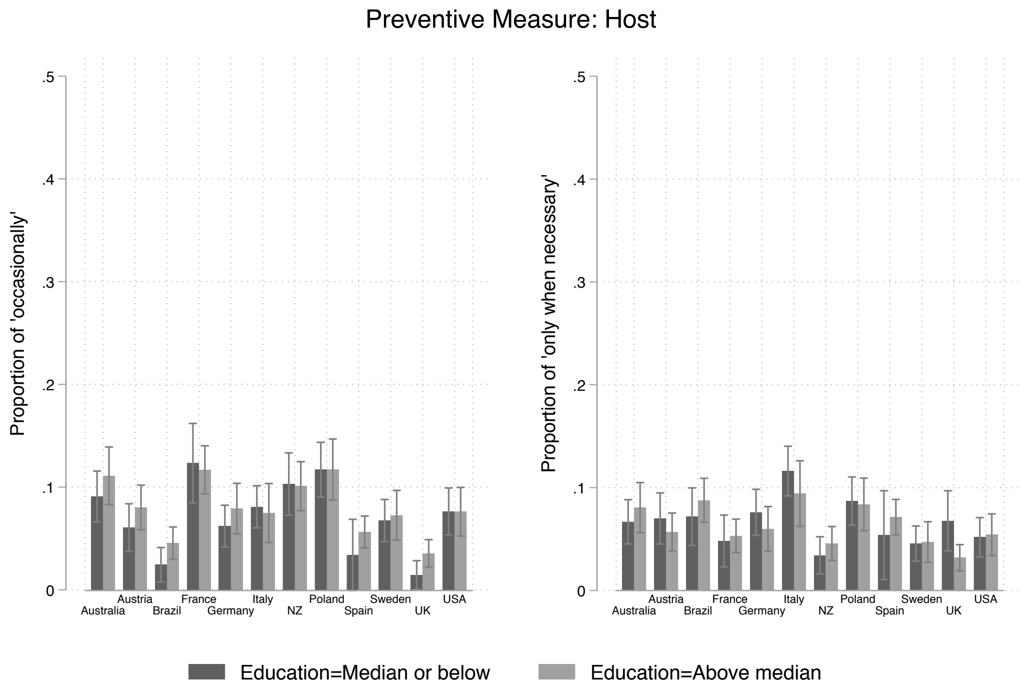

Figure R.4. Distributions of guilt-free answer choices among the least and most educated, across countries: 'No distance' item

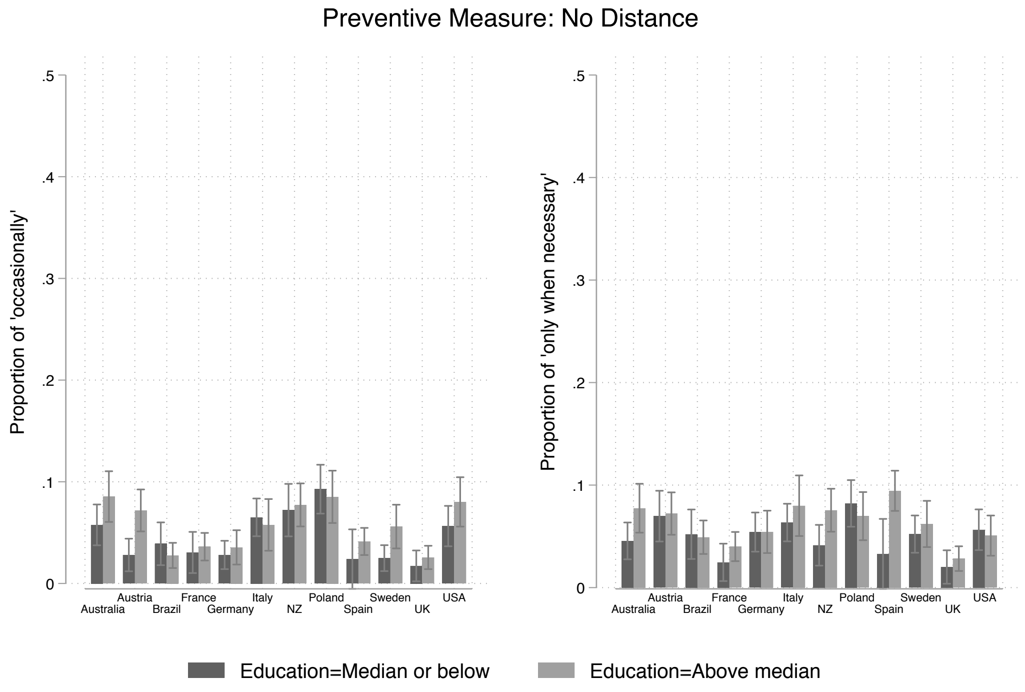

Second, the effects that we find are substantively important, implying that the effect would still be different from zero even if there was a substantial proportion of random answers. Our effects are about 9, 12, 14 and 16 percentage points (Figure 2). Beyond statistical significance (which is reached 47/48 times as shown in Figure 2), these effect sizes are very important. As a result, even if we accept that a subset of the sample, for example 10% of the respondents, answer randomly, the effects would still remain substantial. We agree that the probability of being non-compliant is greater in our guilt-free treatment (as mentioned by R1, it is .75 versus .5), so a 10% of the sample times 25% greater probability would imply that we overestimate the treatment effect by .025 on a 0 to 1 scale. The effect sizes would thus be of 6.5, 9.5, 11.5 and 14.5 percentage points.

Third, and importantly, if there was a problematic proportion of respondents providing random answers, we would expect the treatment effect (i.e. an increase in the number of non-compliance answer choices) to be similar regardless of the level of social desirability of the behaviour. Our results, however, suggest that is not the case and show an effect of the treatment only for those items where social desirability is likely at play. To clarify, the four behaviours used in the main analyses do not allow such a test because they are all proscribed given the public health directives. However, a survey from Canada that we mentioned in the previous version of our manuscript did include more items hence allowing us to distinguish some behaviours that were clearly proscribed versus others that were clearly acceptable. Supplemental analyses of these data clarified that the treatment effect appears conditional on the social desirability of different behaviours (dichotomized here in clearly acceptable versus clearly prohibited). More specifically, we do not find an increase in respondents in the treatment group providing a yes answer when surveying them about whether they used public transportation or went shopping, while we did find such effects for the socially desirable behaviors (e.g. wearing face masks). These results strongly suggests that the potential proportion of the sample that might answer randomly does not alter our findings. Given the importance of these results, we have clarified their implications in the revision of our paper. The relevant paragraph now reads as follows:

> "*In a nutshell, our experimental design is based on the assumption that the differences in the proportion of self-reported non-compliance between the direct and the face-saving questions are related to a reduction of incentives to report socially desirable behaviours. While we think this is a very plausible assumption, we also examine the possibility that another mechanism might explain our results (for example the mere change in the number of response options) using a Canadian survey that was in field during the same period as the twelve surveys examined here. In this Canadian survey, we used the same face-saving strategy but with a broader battery of items, 8 instead of 4. This larger battery included behaviours that were not officially prohibited, i.e. where there should be much less social desirability. In panel A of Figure D.4, we show that there is a strong effect of the treatment (about 10 percentage points) for behaviours that the government prescribed, such as wearing a face-mask in public, and that this effect is much less important in panel B (average of about 3 percentage points) for behaviours that perhaps entail a risk, but that the government did not proscribe – such as taking public transportation or shopping for non-essential products. While we do not have these 'placebo' items for twelve countries included in the analyses, this result for Canada is reassuring and increases our confidence*

that the greater proportion of self-reported non-compliance in the face-saving group is not a 'false positive.'"

Reviewer #2

This paper tackles the important issue of correcting for social desirability when eliciting self-reported preventive measures to prevent the spread of COVID-19. The paper is clearly written, concise, well-executed. The data collection effort across 12 countries is impressive, and well harmonized, providing interesting comparisons in both average reported behaviours and the response to the "treatment". The topic is certainly relevant for public policy and very current.

I have one major concern—the fact that the "face-saving" treatment might correct for social desirability but could introduce experimenter demand effects—a minor concern—related to inference—and a few minor comments and suggestions, which I outline below in bullet points.

***We thank R2 for the positive words and helpful review. Below, we detail how we integrated their comments and suggestions.

Major Concern

Experimenter demand effect

The "face-saving" treatment frames the question in such a way that people feel OK reporting a lower level of preventive behaviour. This framing might have two effects: (1) contrast social desirability upward-bias; and (2) introduce an experimenter demand effect downward-bias. The intent of the author is to tackle the first bias, but I am afraid that they are opening the door to the second type of bias. Since there is no objective external report of these behaviours, there is no benchmark to understand how much this "downward treatment effect" is actually correcting for the first upward bias, or going too far and introducing the second downward bias.
Would it be possible to have a (more) objective or external measure of the actual level of (some of) these preventive behaviours? If not for the individuals in the survey, at least on average for these countries? For example, some mobility data collected by google https://www.google.com/covid19/mobility/ or apple https://covid19.apple.com/mobility
Alternatively, recent papers have tried to tackle the issue of experimenter demand effect (see for example de Quidt et al. 2018 and the citations therein): would it be possible to run a small pilot study in one of these countries to show that experimenter demand effect is not an issue?
If none of these alternatives are feasible, I would at least suggest discussing this issue in the paper.

Regardless of whether this additional analysis is performed, I strongly urge the authors to mention in the paper that there is no way of knowing the "real" level of preventing behaviours, and that all of the statistics that are shown in the paper come from self-reports which cannot be validated.

***The reviewer makes a number of important points here, which we address in the revised version of the manuscript.

First, as the reviewer points out, while our goal is indeed to reduce social desirability, the treatments might have other – unintended – effects as well. To quantify exactly how large the effect of social desirability in the survey responses is, we would indeed have to compare reported responses on the surveyed behaviors with objective information about the occurrence of these behaviours. Unfortunately, retrieving an objective and external benchmark to compare citizens' levels of compliance, is extremely difficult, due to a lack of objective data on the behaviours covered in our study. This is true not only for our respondents, but also in terms of general averages in each country (an approach mentioned by R2). The issue comes from the fact that the existing objective measures like the ones on mobility that are mentioned by R2 only relate to very specific preventive measures. Unfortunately, we are not aware of any objective benchmark for the four items analysed in our paper.[1] We now clearly acknowledge this limitation in the new version of our manuscript. We do so when reviewing the study by Daoust et al. (2020) and also when discussing our findings:

> In the literature review: "*In the current context of the COVID-19 pandemic where it is crucial to understand citizens' level of compliance with preventive measures, Daoust et al. (2020) applied the face-saving strategy to analyze a potential social desirability bias. This latter work showed that face-saving strategies can increase the proportion of citizens who self-report non-compliance with a range of public health preventive measures in Canada. They argue that this increase in self-reported non-compliance is a consequence of reduced social desirability. While there is no objective benchmark to compare these estimates, they substantiate this claim by showing that a similar increase in self-reported non-compliance is not observed when the same face-saving strategy is applied to a series of placebo behaviours that are not prohibited (e.g., grocery shopping).*"

> In the discussion section: *"In this research, we tested the face-saving strategy using a survey experiment in twelve countries to examine the benefits of this approach. We replicated the findings of Daoust et al. (2020) and most importantly, did so in a diverse set of contexts, with different countries and political systems, in different stages of the pandemic (deconfinement in most countries), with different levels of infections, etc. Based on four public health preventive measures related to the wearing of face masks, greetings, hosting gatherings at one's home, and social distancing, we found that the face-saving strategy increased the proportion of citizens who readily answer that they did not comply for 47 out of 48 cases. Most importantly, the effects were substantial. They ranged from 9 percentage points (the mask item) to 16 percentage points (hosting at home) and are robust to several additional tests. Given that there is no objective benchmark for the four preventive measures analyzed in our study that are readily accessible for researchers, we must acknowledge that there is no way to compare our estimates with external measures.*

[1] They are, as outlined in the manuscript: Go shopping or take public transportation without a face mask or taking it off during it; Meet friends, family or colleagues greeting them by shaking hands, hugging or kissing; Have a group of friends or family over at your place; Participate in social activities (work, sport, religious ceremony…) without respecting physical distancing.

Altogether, our experimental design and extensive robustness checks bolster the conclusion that the differences we find can be at least partly attributed to a social desirability bias."

Second, we agree that the potential experimental demand effect in particular is important to address, and we now do so notably by referring to the study mentioned by R2 (de Quidt et al. 2018). While we agree with the reviewer that it is important to flag this issue, we are not overly concerned about the role of experimental demand effects in our particular case. The wording of our preamble is "Some people have altered their behaviour since the beginning of the pandemic, while others have continued to pursue various activities. Some may also want to change their behaviour but cannot do so for different reasons" followed by the question "Have you done any of the following activities in the last week?" Hence, the preamble is composed of two sentences. The first one mentions both directions in terms of the population's level of compliance, (some have altered their behaviour /// some have continued to pursue various activities). The second sentence also mentions both sides (some want to change, but some cannot do so). Given this balanced structure, we believe that we are making it less risky for respondents to admit non-compliance without inducing a clear idea of what was the potential desired outcome for the researchers. It might even be very difficult with the two-sentence preamble to deduce what the hypothesis was, which following Quidt et al. (2018), entails much less substantial effect in terms of experimenter demand compared to the treatments where respondents are asked to do a 'favor' for the researchers. Moreover, Mummolo and Peterson (2019) show in several studies that the experimenter demand effects are likely more limited than what is generally assumed, which is reassuring although it does not mean that there is zero risk.

Third, following the reviewer's comment, we supplemented the manuscript and now report the results from an experiment conducted previously in one of the countries included in our dataset, that is, France. We ran a quota-based online survey that was in field from July 9-19th. As nearly all surveys in France, sampling was done using a quota method based on age, gender, occupation, regions and type of residential area. The sample is designed to be representative of the electorate's features in each of the 13 French regions. 10,686 respondents answered our questionnaire that included our face-saving strategy, but also other estimation techniques that aim to address the issue of social desirability: a list-experiment and 3 other direct questions. The same four items included in the survey were:

- Go shopping or take public transportation without a face mask or taking it off during it;
- Meet friends, family or colleagues greeting them by shaking hands, hugging or kissing;
- Have a group of friends or family over at your place without respecting physical distancing;
- Participate in social activities (work, sport, religious ceremony…) without respecting physical distancing.

As indicated, we used four different strategies to address the issue of social desirability. Three approaches focusing on the question wording and answer options, and a list-experimental approach:

- First, a set of 1343 respondents were exposed to the following "dichotomous" question wording discussed by Daoust et al. (2020): "Have you done any of the following activities in the last week?". The response categories were (1) yes, (2) no.
- Second we administered to another group of 1337 respondents the "polytomous" question used by Munzert & Selb (2020) : "How often have you done the following activities in the last week?". The response categories were (1) daily, (2) several times, (3) once, (4) never.
- The third type of questions included is the "face-saving" question proposed by Daoust et al. (2020): "Some people have altered their behaviour since the beginning of the pandemic, while others have continued to pursue various activities. Some may also want to change their behaviour but cannot do so for different reasons. Have you done any of the following activities in the last week?". 1345 respondents were to choose between the following categories: (1) Yes, (2) Occasionally, (3) Only when necessary, (4) No.
- Finally, we also embedded a list experiment (Becher 2020; Larsen 2020; Munzert & Selb 2020) in this survey. Faced with a list of items, a fourth group of respondents were asked how many things they had done last week, but not which specific ones. Respondents were randomly assigned to either the control group (N=1334) or one of the four treatment groups (N=1332; N=1331; N=1335; N=1329). The treated groups received an additional item capturing the violation of the social distancing norm. The control group received a list of four behaviours that are generally permissible under existing health recommendations. The question reads as follow: "How many of these things have you done last week? You do not need to tell me which ones you have done, just how many". The following four activities were listed: I went to the doctor or to the hospital; I watched a movie or TV show online using a streaming service (e.g., Amazon, Netflix); I exercised outdoors; I ordered food using an online delivery service. In addition to these four items, the treated groups received one of the four more sensitive items we study. The estimated level of non-compliance was obtained from the simple differences-in-means.

Turning to the results from these different approaches, as shown in Figure R.6., for 3 out of 4 items the level of non-compliance estimated by the face-saving question (the estimates denoted by F.S.) is in the 95% confidence interval of the level of non-compliance estimated by the list experiment (the estimates denoted by L.E.). For one item, the face-saving estimate is 4.5 points below and for both other ones it is 2 points higher. For the items related to mask-wearing, greetings and social-distancing in activities, estimates from the dichotomous questions are systematically below the list-experiment estimates and beyond its 95% confidence intervals. The "face-saving" treatment does not seem to induce a systematic experimenter demand effect that would inflate non-compliance, at least compared with an alternative measurement strategy - list experiment – that is well known for decreasing desirability bias (Blair and Imai 2012) and does not appear prone to any experimenter demand effect due to the lack of any associated framing.

The pattern described above differs only for non-compliance in social distancing while hosting friends. In this instance, the list experiment estimate is the lowest. Only the estimate from the dichotomous question does not statistically differ from it. Both the estimates of the face-saving and polytomous questions are significantly higher. The polytomous question is devoid of any framing in the wording of the question. Therefore, even if we have no explanation for this idiosyncratic pattern, it is unlikely that the higher estimate produced by the face-saving question

relative to the list experiment is due to a potential experimenter demand effect induced by the face-saving treatment.

Overall, even if this experiment only includes France, it brings no evidence of an experimenter demand effect that would systematically bias our estimates.

Figure R.6. Predicted level of non-compliance with different items and estimation strategies

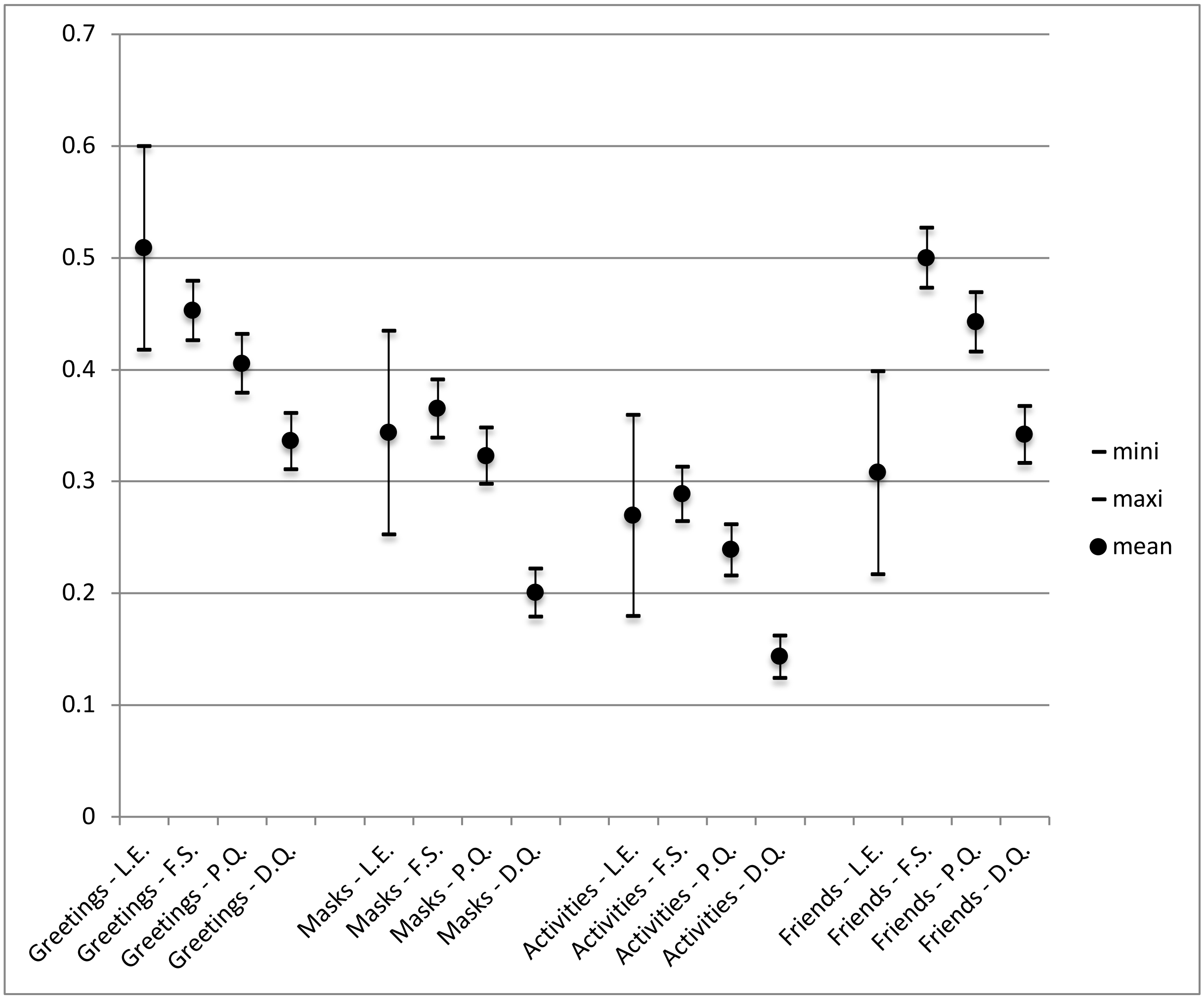

Note: Means with 95% confidence intervals are shown. D.Q. indicates estimation from the "dichotomous" question format; P.Q. indicates estimation from the "polytomous" question format; F.S. indicates estimation from the "face saving" question format; L.E. indicates estimation from the list-experiment estimation strategy. Masks refers to the following items: Go shopping or take public transportation without a face mask or taking it off during it; Greetings refers to the following items: Meet friends, family or colleagues greeting them by shaking hands, hugging or kissing; Activities refers to the following items: Participate in social activities (work, sport, religious ceremony…) without respecting physical distancing. Friends refers to the following items: Have a group of friends or family over at your place without respecting physical distancing.

We now include the following in the new version of the manuscript, as well as the details mentioned in our response to R2 that we include in the Appendix (right after the new Figure):

> "*Fifth, we also check whether or not a potential experimenter demand effect (de Quidt et al. 2018) might drive the results using data from a survey experiment in France. The design of the experiment is presented in the Supplementary Material. Beyond both question formats analyzed earlier, we rely on a list experiment (and a question used by Munzert & Selb (2020)) to estimate the levels of compliance with preventive behaviors from alternative measurement strategies. Results are shown in Figure D.5 of the Supplementary Material. The list experiment approach was designed to reduce social desirability bias and is usually employed for this purpose (Blair and Imai 2012). Most importantly, the risk that there would be an experimenter demand effect is very limited. Overall, even if this experiment only includes one country, using estimates from the list experiment as a baseline brings no evidence of an experimenter demand effect associated to the guilt-free question format that would systematically bias our estimates.*"

Minor Concern

(2) Confidence Interval. I believe that reporting 84% confidence intervals is misleading. I understand that Macgregor-Fors & Payton 2013 suggest it for "visual inspections" of overlapping confidence intervals, but one should NOT consider overlapping CI as a test of difference between plotted coefficients. I would strongly suggest reporting always and only 99% and/or 95% confidence intervals.

***We thank R2 for this comment. We removed 84% CI and focus on 95% ones.

Also, given the number of countries and behaviours reported, it would be nice to have a test correcting for multiple hypothesis testing, in the vein of Romano and Wolf (2005, 2017)---for example, a stata command that implements it is available here https://ideas.repec.org/c/boc/bocode/s458276.html and an R command here https://rdrr.io/github/grayclhn/oosanalysis-R-library/man/stepm.html

***We thank the reviewer for this suggestion. Revising our manuscript, we now include such a test. See below for the mention of this test, as well as the Tables that we included in the Supplementary Material:

> "*Sixth, we implemented the Romano-Wolf correction given the number of tests that we presented (Clarke et al. 2020; Romano & Wolf 2017). Table D.2-5 show that the differences between the treatment and the control group are systematically (45/48) different even using Romano-Wolf* p-*values.*"

Table D.2. Romano-Wolf test for the "No Mask" item

	Model p-value	Resample p-value	Romano-Wolf p-value
Australia	0.0039	0.0050	0.0200
Austria	0.0156	0 0230	0.0509
Brazil	0.0000	0.0010	0.0010
France	0.0002	0.0020	0.0030
Germany	0.0074	0.0060	0.0320
Italy	0.0000	0.0010	0.0010
New Zealand	0.0600	0.0619	0.1249
Poland	0.0000	0.0010	0.0010
Spain	0.0001	0.0010	0.0030
Sweden	0.0000	0.0010	0.0020
United Kingdom	0.1295	0.1518	0.1518
United States	0.0000	0.0010	0.0010

Note: Default options from the rwolf *package was used, with the number of repetitions set at 1000.*

Table D.3. Romano-Wolf test for the "Greeting" item

	Model p-value	Resample p-value	Romano-Wolf p-value
Australia	0.0000	0.0010	0.0010
Austria	0.0071	0.0080	0.0210
Brazil	0.0000	0.0010	0.0010
France	0.0004	0.0010	0.0010
Germany	0.0001	0.0010	0.0010
Italy	0.0000	0.0010	0.0010
New Zealand	0.0002	0.0010	0.0010
Poland	0.0000	0.0010	0.0010
Spain	0.0132	0.0150	0.0260
Sweden	0.0004	0.0010	0.0010
United Kingdom	0.0380	0.0360	0.0360
United States	0.0001	0.0010	0.0010

Note: Default options from the rwolf *package was used, with the number of repetitions set at 1000.*

Table D.4. Romano-Wolf test for the "Host" item

	Model p-value	Resample p-value	Romano-Wolf p-value
Australia	*0.0000*	*0.0010*	*0.0010*
Austria	*0.0035*	*0.0050*	*0.0050*
Brazil	*0.0000*	*0.0010*	*0.0010*
France	*0.0001*	*0.0010*	*0.0020*
Germany	*0.0000*	*0.0010*	*0.0010*
Italy	*0.0000*	*0.0010*	*0.0010*
New Zealand	*0.0000*	*0.0010*	*0.0010*
Poland	*0.0000*	*0.0010*	*0.0010*
Spain	*0.0002*	*0.0010*	*0.0030*
Sweden	*0.0000*	*0.0010*	*0.0010*
United Kingdom	*0.0000*	*0.0010*	*0.0010*
United States	*0.0000*	*0.0010*	*0.0010*

Note: Default options from the rwolf *package was used, with the number of repetitions set at 1000.*

Table D.5. Romano-Wolf test for the "Social Distance" item

	Model p-value	Resample p-value	Romano-Wolf p-value
Australia	*0.0000*	*0.0010*	*0.0010*
Austria	*0.0000*	*0.0010*	*0.0010*
Brazil	*0.0000*	*0.0010*	*0.0010*
France	*0.0000*	*0.0010*	*0.0010*
Germany	*0.0000*	*0.0010*	*0.0010*
Italy	*0.0000*	*0.0010*	*0.0010*
New Zealand	*0.0000*	*0.0010*	*0.0010*
Poland	*0.0000*	*0.0010*	*0.0010*
Spain	*0.0000*	*0.0010*	*0.0010*
Sweden	*0.0000*	*0.0010*	*0.0010*
United Kingdom	*0.0000*	*0.0010*	*0.0010*
United States	*0.0000*	*0.0010*	*0.0010*

Note: Default options from the rwolf *package was used, with the number of repetitions set at 1000.*

*** Questions / Comments / Suggestions ***

• I find the terminology "face-saving strategies" a bit confusing in the setting of the COVID-19 pandemic: when I first read the title, I thought that the authors were referring to wearing a mask (I confused it with "face-covering") or some other form of preventive behaviour (which could be "saving" lives). I would suggest something like "plausible deniability" or "guilt-free". However, this is a purely personal comment, and need not be taken into consideration if the authors do not agree.

***We thank R2 for this comment. We agree that given the current context about the face-mask, our label might be confusing. As suggested by the reviewer, we now use "guilt-free" strategy in the title, as well as in the manuscript. However, we should mention that we still use the term face-saving in our review of the literature, because others as well have used the term, and use it as synonyms, but we believe that changing the title clarifies things.

• For the figures, I would suggest ordering the countries from highest to lowest reported behaviour, and maybe plot them vertically. See for example Cohn et al. (2019).

***We see the merits of the proposed strategy. Doing so would allow to better compare the values across countries for a given preventive measure. However, the countries' position on the x-axis would change, which would prevent the reader from easily spot a given country across the four items. Hence, we kept our initial visualization.

• The graphs and tables have only minimal notes, it would be useful for the cursory reader to have more information available right at the bottom of the graph

***We thank R2 for the comment and we now include more information as notes to the Tables and Figures, including the ones in the online appendix.

• It could be helpful to have a table or a list of the behaviours that are allowed/suggested/prohibited in the different countries at the time of the data collection, or some form of stringency of the measures used by each country, similar to https://www.bsg.ox.ac.uk/research/research-projects/coronavirus-government-response-tracker#data or https://ourworldindata.org/grapher/covid-stringency-index

***This is a great suggestion and we now included a Table that adds more context to the cases that can be found in the Supplementary Material. The reader will now have detailed information about the data for each country, the death rates, and the preventive measures in place. In the revision of our paper, we refer to this table in the following way:

> "*Table A.1 of the Supplementary Material lists the exact dates of data collection in each country, as well as the number of observations. Moreover, Table A.2 shows the population per country and the death rates (per 100 000 inhabitants) as of June 15th and Table A.3. provides an overview of the preventive measures that were recommended and compulsory in each country*." We copied the new Table A.3. below.

Table A.3. COVID-19 Preventive Measures across countries (on day 1 of the data collection for each country).

	Mandatory use of masks in shops and public transportation		Mandatory physical distancing		Stay at home requirement	
	Policy	Scope	Policy	Scope	Policy	Scope
**Australia**	No	National	Yes	National	No	National
**Austria**	In public transportation	National	Yes	National	No	National
**Brazil**	In shops and/or public transportation	In specific areas in state or in whole state	Yes	In specific areas in state or in whole state	Require not leaving house with exceptions for daily exercise, grocery shopping, and 'essential' trips	In specific areas
**France**	In public transportation	National	Yes	National	No	National
**Germany**	In public transportation and shops	National (agreement of federal and state governments) / all states except Berlin	Yes	National	No	National
**Italy**	In shops and public transportation	National	Yes	National	Recommend not leaving house	National
**New Zealand**	No	National	No	National	No	National
**Poland**	In shops and public transportation	National	yes	National	No	National
**Spain**	In shops and restaurants; also in all public spaces when social distancing not possible	Specific regions / national	Yes	National	Recommend not leaving house	In specific regions
**Sweden**	No	National	No	National	Recommend not leaving house	National
**United Kingdom**	In public transportation	National	Yes	National	Recommend not leaving house	In specific areas
**United States**	In shops and/or public transportation	In specific areas in state or in whole state	Yes	In specific areas in state or in whole state	Require not leaving house with exceptions for daily exercise, grocery shopping, and 'essential' trips	In specific areas in state or in whole state
**Canada***	For air passengers / in shops and public transportation	National / in some provinces or in specific areas of some provinces	Yes	National	Recommend not leaving house	National

Source: https://github.com/amel-github/covid19-interventionmeasures; https://masks4all.co/what-countries-require-masks-in-public; https://airtable.com/shrxEdbaBJIQwMUgA/tbl5o6qUd54BL9wkw; https://www.bsg.ox.ac.uk/research/research-projects/coronavirus-government-response-tracker; https://kubinec.shinyapps.io/coronanet; and further authors' corroborations with official and media sources.

• The precise definition of the treatment comes only at the bottom of page 7. It might be useful for the reader to have it earlier, maybe even in the introduction.

***We agree with R2 and we are thankful for the suggestion. We now include more details about the treatment in the introduction. The revised paragraph now reads as follows:

> "*When exposed to a short preamble (referring to the fact that some people have altered their behaviours since the beginning of the pandemic, while others have continued to pursue various activities) combined with guilt-free answer choices (including "only when necessary"), respondents in the Daoust et al. (2020) study were substantially more likely than respondents from the control group to report non-compliant behaviour in the context of the COVID-19 pandemic*."

Moroever, we kept the full description of the treatment in the method section:

> *"Here, we make use of the most effective face-saving strategy identified by Daoust et al. (2020), which is also the strategy they recommend for future work (their Study 3). Extending this work to other countries, half of each national sample was randomly allocated a direct question while the other half received the face-saving treatment. The direct question was: "Have you done any of the following activities in the last week?" followed by a set of four items and yes/no answer choices.*[2] *The face-saving question preamble was*:
>
> *Some people have altered their behaviour since the beginning of the pandemic, while others have continued to pursue various activities. Some may also want to change their behaviour but cannot do so for different reasons. Have you done any of the following activities in the last week*?"

References:

Clarke, D., Romano, J. P., & Wolf, M. (2020). The Romano-Wolf Multiple Hypothesis Correction in Stata. IZA Working Paper. https://www.iza.org/publications/dp/12845/the-romano-wolf-multiple-hypothesis-correction-in-stata

Romano, J. P., & Wolf, M. (2017). Resurrecting weighted least squares. Journal of Econometrics, 197(1), 1–19. https://doi.org/10.1016/j.jeconom.2016.10.003

Romano, J. P., & Wolf, M. (2005). Exact and Approximate Stepdown Methods for Multiple Hypothesis Testing. Journal of the American Statistical Association, 100(469), 94–108. https://doi.org/10.1198/016214504000000539

Cohn, A., Marechal, M. A., Tannenbaum, D., & Zünd, C. L. (2019). Civic honesty around the globe. Science, eaau8712. 2

[2] Respondents could skip the question. Less than 0.5% did so in every country.

de Quidt, J., Haushofer, J., & Roth, C. P. (2018). Measuring and Bounding Experimenter Demand. American Economic Review, 108(11), 3266–3302. https://doi.org/10.1257/aer.20171330

Reviewer #3

A very nice paper, well written, very transparent. My only concern is, that their claim of using a "new strategy" is a bit overstretched. Below I give more details on that criticism. Still I am convinced that the paper is a valuable contribution to the sensitive question literature and - with its focus on surveying adherence to covid preventive measures - is highly relevant.

• What are the main claims of the paper and how significant are they for the discipline?
The authors replicate a previous study about the effect of face-saving strategies on self-reports of non-compliance when surveying compliance to covid-19 measures (e.g. social distancing). They ran the experiment in twelve countries and find consistent evidence of a positive effect of the strategy under investigation. The topic is highly relevant because it tackles a survey methodology issue that is of utmost importance under the actually circumstances (the pandemic).

***We thank R3 for their helpful and positive review. Below, we detail how we revised our manuscript in order to incorporate their comments.

• Are the claims properly placed in the context of the previous literature? Have the authors treated the literature fairly? In general yes. My only criticism is, that what they present as “new face-saving strategy” is basically simply choosing likert-scale response categories instead of dichotomous yes/no response option. In addition, only providing the yes or no response options are for most of the surveyed behaviours such as keeping distance, wearing masks, obviously not very suitable. I would guess, that not much researcher have used "primitive" yes/no response options for surveying adherence to such measures.

Still, I find it valuable to have a close look at this topic and to study how response options influence answer behavior. But the authors should explain a bit more detailed in what regard their strategy is really new (or not so much).

***This is a fair point. We should make it clear that the face-saving strategy, although not always labeled as such, has been previously used in the study of various topics. We revised the manuscript accordingly. In the abstract, we removed the “proposed by Daoust et al. (2020)” and replaced it by “used by Daoust et al. (2020).” Moreover, we include new examples from different disciplines in the literature review. See below:

> *“Such an approach has been applied to a wide range of topics. For example, political scientists used it to study voter turnout (Belli et al. 2006; Morin-Chassé et al. 2017) where there is a clear norm that voting is the right thing to do. It has also proven to be useful to study illicit behavior like shoplifting (Holtgraves et al. 1997), sexual/health behaviour such as using a condom during sexual intercourse (Catania et al. 1996; Raghubir and Menon 1996), or consumer choice in market research (Nancarrow et al. 2001).*

In the current context of the COVID-19 pandemic where it is crucial to understand citizens' level of compliance with preventive measures, Daoust et al. (2020) applied the face-saving strategy to analyze a potential social desirability bias."

• I also wonder, whether the authors could provide information about the effect of the response option and the question preamble separately. In their design these two aspects are combined.

***This is a good point. We did not test every combination possible as our primary goal in this study was to test the generalizability of the particular face-saving approach recommended in Daoust et al. 2020. To be sure, Daoust et al. (2020: 6-7) were quite clear about the findings using alternative design (for example, only the preamble). They mention that "Our research design applied to our three studies does not allow us to fully disentangle the relative effects of the face-saving prompt (i.e. the preamble) from the guilt-free options, and as a result, we cannot definitively conclude that a guilt-free option would be sufficient to obtain more accurate measures of non-compliance. We can conclude from Study 1 that the preamble alone is not sufficiently strong to reduce social desirability. However, it is not impossible that the guilt-free answer choices are only effective in combination with a face-saving preamble." This is why we use both the preamble and the guilt-free answer choices. Although we could have conducted a new experiment to assess whether or not the guilty-free answer choices are sufficient or conditional on the preamble, this was not our goal in this particular paper, and something we leave for future work.

• Another issue is, that their strategy is only applicable to sensitive behaviours whose sensitivity is somehow "gradual" in nature. E.g. it works for shaking hands or wearing face masks. But it wouldn't work for having been arrested, committing tax fraud, or voting for a right-wing party. I would wish, authors would comment on that.

***This is a fair point. We now provide more context about the use of the face-saving strategy and how it can be used. We notably included more examples of how the approach was used across different disciplines as mentioned above (illicit behaviour like shoplifting, sexual behaviour and the use of condoms, voter turnout, and consumer choices). We believe that it is possible to create several response options to alleviate social desirability in many other contexts.

We believe that the design could be applied to other topics, including tax evasion (mentioned by R3). For example, there could be a preamble cueing that some people sometimes avoid paying taxes or do so without knowing that what they are doing is illegal. Answer choices could also complement this guilt-free approach by moving from a binary answer choice to something like "Once" / "Twice or more" / "Unintentionally only" / "Never."

• Do the data and analyses fully support the claims? If not, what other evidence is required? Yes. Design and analysis are reported very carefully and transparently. Authors provide various robustness checks, analysis by countries, additional details, and consider, for instance, the threat of "false positives" that might invalidate their findings.

***We thank R3 for the review that helped us to strengthen our manuscript and hope that they will find and answers satisfying.

New References

Anduiza, E., & Galais, C. (2017). Answering without reading: IMCs and strong satisficing in online surveys. *International Journal of Public Opinion Research*, *29*(3), 497-519.

Belli, R.F., Moore, S. E., VanHoewyk, J. (2006). An experimental comparison of question forms used to reduce vote overreporting. *Electoral Studies*, 25(4): 751-759.

Blair, G., & Imai, K. (2012). Statistical Analysis of List Experiments. *Political Analysis, 20*(1), 47-77. doi:10.1093/pan/mpr048

Catania, J. A., Binson, D., Canchola, J., Pollack, L. M., Hauck, W., & Coates, T. J. (1996). Effects of interviewer gender, interviewer choice, and item wording on responses to questions concerning sexual behavior. *Public Opinion Quarterly*, *60*(3), 345-375.

Galasso, V., Pons, V., Profeta, P., Becher, M., Brouard, S., and Foucault, M. (2020). Gender differences in COVID-19 attitudes and behavior: Panel evidence from eight countries. *PNAS*, 117 (44): 27285-27291.

Holtgraves, T., Eck, J. and Lasky, B. (1997). Face Management, Question Wording, and Social Desirability. *Journal of Applied Social Psychology*, 27: 1650-1671.

Maniaci, M. R., & Rogge, R. D. (2014). Caring about carelessness: Participant inattention and its effects on research. *Journal of Research in Personality*, *48*, 61-83.

Mummolo, J., & Peterson, E. (2019). Demand effects in survey experiments: An empirical assessment. *American Political Science Review*, *113*(2), 517-529.

Nancarrow, C., Brace, I. and Wright, L. T. (2001). "Tell me Lies, Tell me Sweet Little Lies": Dealing with Socially Desirable Responses in Market Research. *The Marketing Review*, 2(1): 55-69.

Raghubir, P., & Menon, G. (1996). Asking sensitive questions: The effects of type of referent and frequency wording in counterbiasing methods. *Psychology & Marketing*, *13*(7), 633-652.
